# Proline Isomerization Regulates the Phase Behavior of Elastin-Like Polypeptides in Water

**DOI:** 10.1021/acs.jpcb.1c04779

**Published:** 2021-08-23

**Authors:** Yani Zhao, Kurt Kremer

**Affiliations:** Max Planck Institute for Polymer Research, Ackermannweg 10, 55128 Mainz, Germany

## Abstract

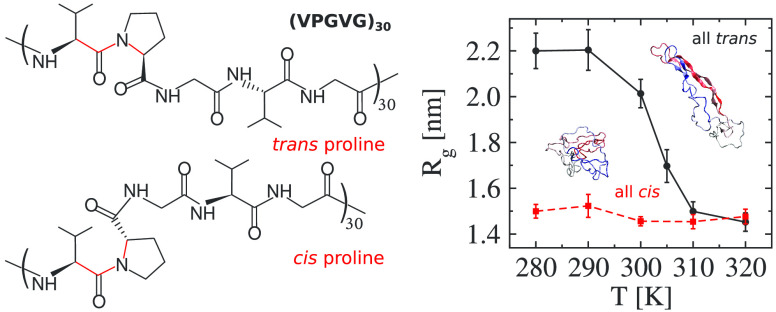

Responsiveness of polypeptides and polymers in aqueous solution plays an important role in biomedical applications and in designing advanced functional materials. Elastin-like polypeptides (ELPs) are a well-known class of synthetic intrinsically disordered proteins (IDPs), which exhibit a lower critical solution temperature (LCST) in pure water and in aqueous solutions. Here, we compare the influence of *cis*/*trans* proline isomerization on the phase behavior of single ELPs in pure water. Our results reveal that proline isomerization tunes the conformational behavior of ELPs while keeping the transition temperature unchanged. We find that the presence of the *cis* isomers facilitates compact structures by preventing peptide–water hydrogen bonding while promoting intramolecular interactions. In other words, the LCST transition of ELPs with all proline residues in the *cis* state occurs with almost no noticeable conformational change.

## 1 Introduction

Stimulus-triggered polypeptides are involved in a wide range of biological processes. For example, the liquid–liquid phase separation of intrinsically disordered proteins (IDPs) is found to contribute to the formation of membraneless organelles,^1,2^ and the self-assembly of IDPs is associated with numerous human diseases^3,4^ including neurodegenerative disorders, cancer, and amyloidoses. Synthetic polymers that exhibit phase transitions also have broad applications ranging from biomedical applications^5−9^ to polymer materials design.^10−15^ Therefore, the microscopic understanding of the phase behavior of stimuli responsive polymers is crucial for the optimized future applications.^14,16^

Elastin-like polypeptides (ELPs)^16,17^ are synthetic peptide-like polymers with pentapeptide repeat sequences Val-Pro-Gly-Xaa-Gly (VPGXG), where the guest residue Xaa can be any amino acid except proline. They typically exhibit a lower critical solution temperature (LCST) phase behavior in aqueous solution, with an expanded-to-collapsed conformational transition. The transition temperature *T*_*l*_ of ELPs is tunable and depends on the peptide sequence, the chain length,^18^ and a number of external stimuli, such as changes in pH,^19^ ion concentration,^20^ and pressure.^21^

**Figure 1:**
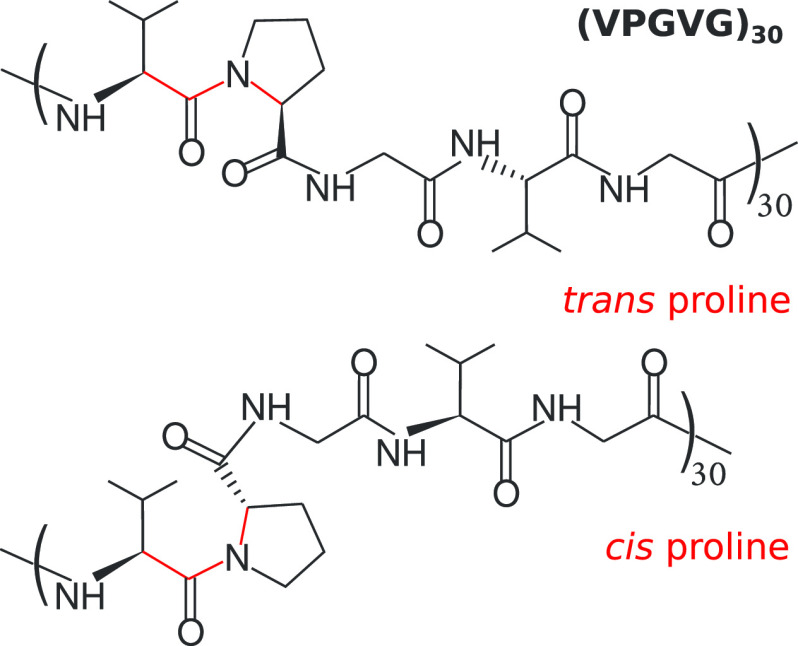
Schematic representation of (VPGVG)_30_ with *trans* or *cis* proline isomers. The backbone residues which restrict the ω dihedral angle of the Val–Pro amide bonds are marked in red; ω = 180° for the *trans* isomer, while it is 0° for the *cis* isomer.

ELPs are proline-rich peptides; however, the effects of proline isomerization on their phase behavior remain unclear. Proline is the only amino acid with a cyclic side group; i.e., its nitrogen atom is linked to two carbon atoms, forming a five-membered ring (see Figure 1). This unique structure stabilizes both *cis* and *trans* isomers. While the Gibbs free energy difference between the two proline isomers is only ∼2 *k*_B_*T*,^22,23^ their transition barrier is rather high, ∼30–32 *k*_B_*T*.^22,24^ Therefore, proline isomerization is a fairly slow rate-limiting process,^25^ which is important in understanding protein folding kinetics.^26^ In nature, the *trans* isomer is dominant in Xaa–Pro peptide bonds with a *trans*:*cis* ratio^27^ of about 88:12, in excellent agreement with a direct Boltzmann weight energy based estimate. However, one can enhance the *cis* isomer content in a number of ways including replacing a proline with a pseudoproline named ΨPro,^25^ using proline isomerase assay^22^ or the C(4)-position substituent,^28^ and also perhaps by ultraviolet photodissociation.^29^

In this work, we focus on the effect of proline isomerization on the phase behavior of ELPs using all-atom simulations. We consider an ELP sequence of (VPGVG)_30_ with four different *cis* proline compositions: (i) all proline residues are in the *trans* state *P*_*cis*_ = 0; (ii) half of the proline residues are in the *cis* state (*P*_*cis*_ = 0.5), and they are either organized in two blocks *cccccccccc cccccttttt tttttttttt* or (iii) ideally mixed, *ctctctctct ctctctctct ctctctctct*; and (iv) all of the proline residues are in the *cis* state (*P*_*cis*_ = 1.0). Note that these are model sequences to best isolate the effect of the *cis* isomers. To simplify the notation, these four cases will be denoted as all-*trans*, hs-*cis*, hm-*cis*, and all-*cis*, respectively, in the following text. Because of the high energy barrier, the *trans*/*cis* composition remains constant during the course of the simulation. Our results show that proline isomerization plays an important role in tuning the conformational behavior of ELPs in water while keeping *T*_*l*_ unchanged. The presence of the *cis* isomers facilitates rather compact structures of the peptide. These structures remain largely stable in the temperature range studied, because of enhanced intramolecular and reduced peptide–water hydrogen bonds. The compactness of the peptide is a function of both the percentage and the position of *cis* isomers. The more *cis* isomers and the more distributed they are along the sequence, the more compact the chains are.

Our study reveals that the conformational behavior of ELPs and other proline-rich peptides can be regulated by proline isomerization while keeping their transition temperature unchanged. To demonstrate this most clearly, we especially focus on the all-*trans* and all-*cis* cases.

## 2 Methods

The all-atom simulations of (VPGVG)_30_ were performed using the GROMACS molecular dynamics (MD) package,^30^ where the initial structures were prepared with the PyMOL package^31^ (see Figure S1a,b in the Supporting Information). The MD simulations were performed in the *NPT* ensemble, using the CHARMM36m force field^32^ together with the TIP3P water model.^33^ The pressure was kept at 1 bar using the Parrinello–Rahman–Andersen barostat^34^ with a coupling constant of 2 ps, and the temperature of the system was kept constant by a velocity rescaling thermostat^35^ with a coupling constant of 1 ps. The electrostatic interactions were simulated using the particle mesh Ewald (PME) algorithm.^36^ The cutoff of the electrostatic and van der Waals interactions was set to 1.4 nm. We used the LINCS algorithm^37^ for bond constraints. The equations of motion were integrated using the leapfrog integrator with a time step of 2 fs.

The chosen ELP with sequence (VPGVG)_30_ is a relatively well studied system.^38,39^ Its transition temperature was computationally estimated^18^ (there the atomistic simulations were performed using *Amber 11* with ff99SB force, which might lead to slightly different transition temperatures) as *T*_*l*_ = 307.5 ± 2.5 K. For such a short chain, one cannot expect a sharp phase transition in the simulations. Therefore, we considered the simulation temperature range from *T* = 280 to 320 K to take into account the region around its LCST transition. In our simulations, the peptide was hydrated in a 10 nm × 10 nm × 10 nm box with 32,177 water molecules. We used the replica exchange molecular dynamics (REMD) to enhance the sampling of the peptide in the all-*trans* case around its transition temperature. In total, there are five temperature replicas ranging from 300 to 310 K, and the exchange of replicas was attempted every 2 ps. The REMD simulations lasted for 1 μs. In other cases, we generated two independent trajectories for each temperature, and these trajectories covered a time of 1 μs.

The geometry of the peptide with *cis* or *trans* proline isomers was characterized by measuring the ω dihedral angle of the Val–Pro amide bonds and the effective backbone length . Here, **r**_*i*_ corresponds to the position of the C_α_ atom of the *i*th residue along the backbone of (VPGVG)_30_ and *N* = 150 is the total number of residues. Because of the high energy barrier between the *cis* and *trans* states, no *cis*/*trans* transition was observed in the course of our simulations for a given case. The dimension of the peptide was characterized by the gyration radius , the end-to-end distance , the solvent-accessible surface area (SASA),^40^ and the single-chain backbone structure factor , where **q** is the wave vector. Here, the calculation of *S*(*q*) is based only on the Cα atoms. The secondary structural content of the peptide including the propensity of β-sheet and α-helix was estimated by the DSSP algorithm, which assigns secondary structures based on the backbone hydrogen bonds using an electrostatic model.^41^

We also performed a hydrogen bonding analysis by counting both the number of hydrogen bonds (H-bonds) between the peptide and water molecules *N*_pw_ and the number of the intramolecular H-bonds within the peptide *N*_pp_. Additionally, we counted the H-bonds formed between proline and the non-proline residues *N*_pro,np_ to characterize the effects of *cis*/*trans* proline isomerization. H-bonds are estimated using the standard GROMACS subroutine; i.e., an H-bond exists if the donor–acceptor distance is ≤0.35 nm and the acceptor–donor-hydrogen angle is ≤30°. To characterize the density of water molecules within distance *r* from the peptide, we calculated the radial distribution function between the backbone of the peptide and the oxygen atoms on the water molecules *g*_pw_(*r*).

## 3 Results and Discussion

**Figure 2:**
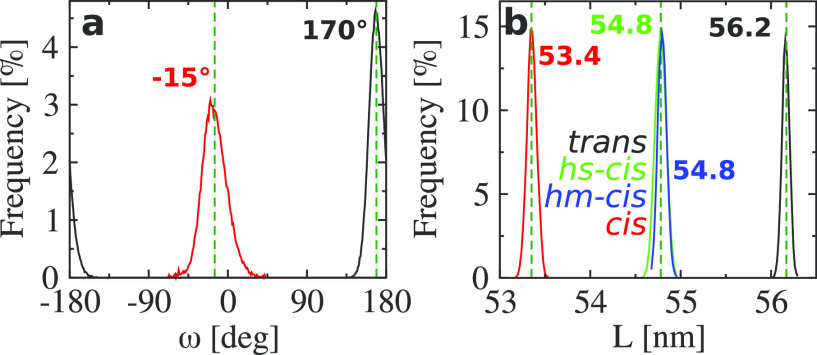
(a) The comparison of the ω dihedral angle between the *trans* and *cis* isomers at *T* = 280 K. The values of ω at other studied temperatures can be found in Figure S1. (b) The effective backbone length *L* of (VPGVG)_30_ in the all-*trans*, hs-*cis*, hm-*cis*, and all-*cis* cases. For a given case, *L* is independent of temperatures.

To characterize the internal structure (backbone orientation) of (VPGVG)_30_ for the four distinct *cis* proline compositions considered, we analyze the dihedral angles, ω, of the Val–Pro amide bonds and the effective backbone lengths *L*. The distribution of ω at *T* = 280 K is shown in Figure 2a. Note that the ω distribution remains the same with temperature; see Figure S1c,d. We find that the average value of ω is 170° for the *trans* Val–Pro bonds, while it is −15° for the *cis* bonds, roughly consistent with the ideally expected difference of 180° between *trans* and *cis* isomers. This small deviation in ω is expected because of the local bending and packing interactions within a molecule. In the cases of hs-*cis* and hm-*cis*, the distribution of ω in the region with *trans* isomers is the same as that from the all-*trans* case, while that with *cis* isomers is the same as that for the all-*cis* case.

Figure 2b shows the distribution of *L* along with the average values ⟨*L*⟩ = 56.2, 54.8, 54.8, and 53.4 nm in the cases of all-*trans*, hs-*cis*, hm-*cis*, and all-*cis*, respectively. The difference of ⟨*L*⟩ between the all-*trans* and all-*cis* cases is Δ*L* = ⟨*L*_*trans*_⟩ – ⟨*L*_*cis*_⟩ = 2.8 nm, which gives an average elongation of ∼0.1 nm per proline. The elongation has also been observed experimentally,^23^ which indicates a similar backbone change of *cis*-to-*trans* isomerization, as shown in our simulations. The results of *L* and ω provide a detailed geometric picture of the peptide; i.e., its internal structure with the *trans* isomers is different than that with the *cis* isomers.

**Figure 3:**
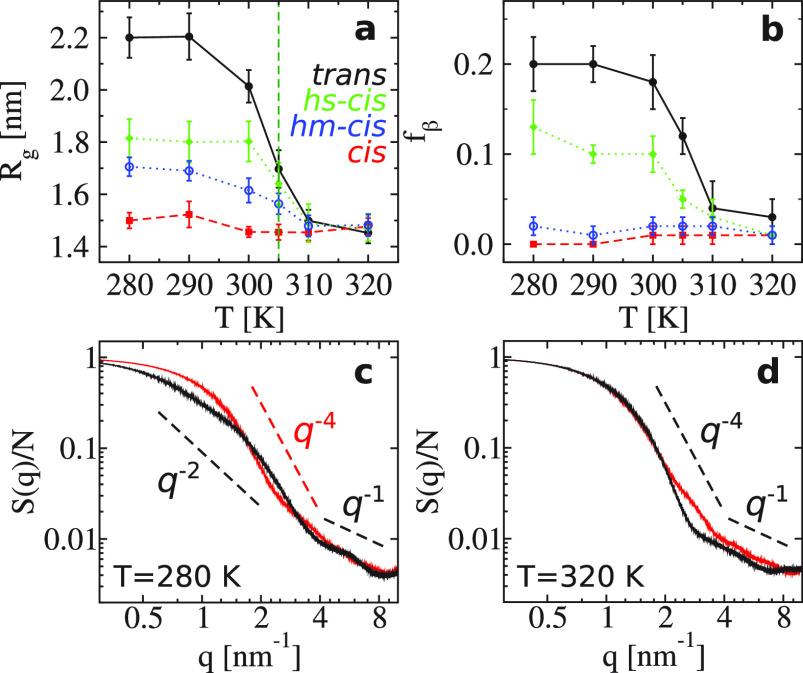
(a, b) The results of *R*_g_ and *f*_β_ of (VPGVG)_30_ in the all-*trans* (black), hs-*cis* (green), hm-*cis* (blue), and all-*cis* (red) cases in pure water. The dashed vertical line in panel a indicates *T*_*l*_ ≃ 305 K detected in our work. (c, d) *S*(*q*) at *T* = 280 and 320 K in the all-*trans* (black) and the all-*cis* (red) cases.

In Figure 3a, we show the effects of proline isomerization on the gyration radius *R*_g_ of the system as a function of temperature. It can be seen from the all-*trans* data that the peptide shows a well-defined expanded-to-collapsed transition upon an increase of temperature; the detected LCST transition temperature (approximate inflection point of the curve) is around *T*_*l*_ ≃ 305 K. The obtained *T*_*l*_ is in very good agreement with experiments, which found *T*_*l*_ = 299 K for the sequence (VPGVG)_*n*_.^20^ At the other extreme, the all-*cis* case, *R*_g_ is nearly independent of temperature; i.e., no LCST-like transition signature in *R*_g_ is observed. In the mixed cases, the size of the peptide is in between the former two. Additionally, the difference between *R*_g_ of hs-*cis* and hm-*cis* illustrates how the structure of the peptide is affected by the sequence of the *cis* isomers along the backbone. In particular, *R*_g_ of hs-*cis* follows a transition similar to the all-*trans* case albeit more attenuated (half is collapsed and half remains expanded when *T* < *T*_*l*_), while that of hm-*cis* is further reduced. Note that *cis* isomers may contribute up to 12% of Val–Pro peptide bonds in nature.^27^ Thus, we have considered an additional system with *P*_*cis*_ = 0.1, close to the natural *cis* content. As shown in Figure S2, 10% *cis* content leads to a 5–13% decrease of *R*_g_ at temperatures below *T*_*l*_, depending on the *trans*/*cis* sequence, while the transition temperature is not affected.

To characterize the local interactions of the peptide, we also estimate the propensity of secondary structure formation. For the sequence (VPGVG)_30_, we expect it to form β-sheets (it has two valine residues in each pentapeptide) but not α-helices (proline and glycine are known to prohibit helix formation). It is indeed the case in the all-*trans* case but not in the all-*cis* case, which also has no β-sheets. Figure 3b presents the results of *f*_β_, the fraction of β-sheets formed by connecting the adjacent β-strands laterally with hydrogen bonds (*f*_β_ is obtained by the DSSP algorithm^41^). We find that *f*_β_ exhibits a similar trend as a function of temperature as *R*_g_ in the four systems. In the all-*trans* case, *f*_β_ decreases monotonically from ∼20 to ∼5% as *T* increases from 280 to 320 K. The temperature induced decrease in *f*_β_ is due to the fact that less β-strands can be laterally placed to form β-sheets as the chain becomes more compact. A similar pattern is seen in the hs-*cis* case with smaller values of *f*_β_ for *T* < *T*_*l*_. A closer look reveals that the decrease is mostly coming from the region with *trans* isomers (see Figures S3 and S4). This observation clearly shows that the presence of the *cis* isomers sterically hinders the formation of hydrogen bonds between local segments, which explains why *f*_β_ is nearly zero at all temperatures in the hm-*cis* and all-*cis* cases. In conclusion, we observe that *T*_*l*_ of the ELP seems independent of the percentage and position of the *cis* isomers (see the vertical line in Figure 3a), yet the amplitude of the *R*_g_ signature and the secondary structural content are strongly dependent on the *cis* composition. For the all-*cis* case, the *R*_g_ signature on the single chain level almost vanishes, requiring further consideration.

The global conformation of the ELP is also well characterized by the backbone structure factor *S*(*q*). *S*(*q*) for the all-*trans* and all-*cis* cases at *T* < *T*_*l*_ (*T* = 280 K) and *T* > *T*_*l*_ (*T* = 320 K) are presented in Figure 3c,d. Results for other temperatures are shown in Figure S5. The *S*(*q*) data show that at *T* = 280 K the all-*cis* chain assumes a globular state (*q*^–4^ scaling), while the all-*trans* chain more closely resembles a random walk structure (*q*^–2^) at *q* values below about 2 nm^–1^ with a more compact regime on shorter length scales. On smaller scales above *q* ≈ 4 nm^–1^, *S*(*q*) is very similar in both cases. At *T* = 320 K, the all-*trans* chains collapse into an even more pronounced globular structure compared to the all-*cis* case, as demonstrated by Figure 3d (*S*(*q*) of the all-*cis* chain remains essentially unchanged from *T* < *T*_*l*_ to *T* > *T*_*l*_). Data for the hs-*cis* and hm-*cis* cases interpolated between these two extremes are shown in the Supporting Information. The power laws indicated by dashed lines are used to guide the eye. Clearly, the peptide behaves roughly similar to a short polymer chain in between the Θ and the collapsed state in the all-*trans* case, and it becomes significantly more compact from the outset as *P*_*cis*_ increases. At high *q* when  ∼ 3 nm^–1^, the scaling of *S*(*q*) transitions to *q*^–1^ in all cases, where the peptide behaves essentially like a rigid rod. These data agree well with the observations obtained from *R*_g_ and *f*_β_. Moreover, the obtained Kuhn length *l*_k_ ∼ 2 nm (the length of 5–6 residues) agrees with our previous simulation^42^ and the experimental results^43^ for ELPs. We also calculated the hydrodynamic radius *R*_h_ of (VPGVG)_30_; data are shown in Table S1 for reference. For ideal chains, one can estimate the end-to-end distance *R*_e_ of the chain directly by the Kuhn length as . Using *L* = 56.2 nm (see Figure 2) and *R*_e_ shown in Table S1 is a clearly too small value for *l*_k_, revealing significant deviations from a Gaussian structure. In general, the data presented here agree quite well with experimental data obtained from dynamic light scattering^43^ (for details of the different radii, we refer to the Supporting Information). The deviations from the classical polymer picture may be due to the fact that the ELP chain has a fraction of ∼20% of β-sheets in the all-*trans* case at *T* < *T*_*l*_. Nevertheless, the applied model can successfully catch the LCST transition of the ELP (Figure 3) and can distinguish between the *cis* and *trans* proline states (Figure 2). Above the LCST, *S*(*q*) displays a *q*^–4^ scaling in all considered cases, which means that the peptide is collapsed regardless of the *cis* content. Note that, although the shape parameters *S*(*q*) and *R*_g_ in all considered cases are alike at *T* > *T*_*l*_, the internal structure (which can be characterized by the ω dihedral angles of the Val–Pro amide bonds and the effective backbone lengths *L*) of the peptide remains very different, as shown in Figure 2.

**Figure 4:**
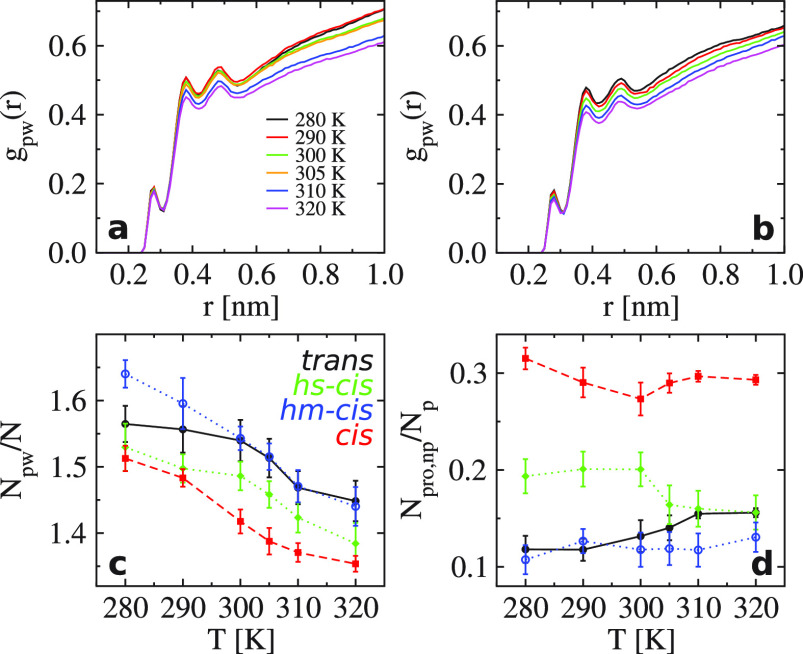
(a) The radial distribution function *g*_pw_(*r*) of (VPGVG)_30_ between peptide atoms and water oxygens in the all-*trans* case as a function of *r* at different temperatures. (b) The same as part a but for the all-*cis* case. (c) Number of H-bonds *N*_pw_/*N* normalized by the total number of residues *N* = 150 in the all-*trans* (black), hs-*cis* (green), hm-*cis* (blue), and all-*cis* (red) cases. (d) Intramolecular H-bonds *N*_pro,np_/*N*_p_ normalized by the number of proline residues *N*_p_ = 30. The color code is the same as that in panel c.

The LCST phase transition (the expanded-to-collapsed transition upon increase of temperature) is entropy driven,^44^ i.e., dominated by the translational entropy gain of the water molecules upon collapse of the peptide around *T* > *T*_*l*_. The amount of released water molecules can be visualized by the radial distribution function.^45^ As shown in Figure 4a,b, *g*_pw_(*r*) of (VPGVG)_30_ has three peaks within *r* ≤ 1.0 nm (note that the correlation length of water molecules is less than 2.0 nm in the considered systems^13^). We find that the height of the peaks decrease as *T* increases, because the peptide becomes more compact (see Figure 3a). However, in the all-*trans* case, a jump of *g*_pw_(*r*) around *T*_*l*_ is observed, while, in the all-*cis* case, no jump but a continuous decrease is observed. In the mixed cases, *g*_pw_(*r*) of hs-*cis* is closer to that in the all-*trans* case, and *g*_pw_(*r*) of hm-*cis* is more similar to that in the all-*cis* case. Interestingly, the amplitude of the three peaks in *g*_pw_(*r*) satisfies *g*_pw,hm-*cis*_(*r*) < *g*_pw,all-*trans*_(*r*) < *g*_pw,hs-*cis*_(*r*) < *g*_pw,all-*cis*_(*r*) at *T* < *T*_*l*_ (an example can be found in Figure S6). The case of hm-*cis* has the largest *g*_pw_(*r*), because it has the largest solvent-accessible surface area (SASA, which measures the surface area of the ELP that is accessible to solvent molecules), compared with the other cases; see Figure S7a,b. These observations are further supported by the results of the peptide–water H-bonds *N*_pw_/*N* shown in Figure 4c (see also Figures S8 and S9); i.e., *cis* isomers prevent the formation of peptide–water hydrogen bonds.

We also calculated the intramolecular H-bonds in all considered cases. Figure 4d shows the results of *N*_pro,np_/*N*_p_, the H-bonds formed between proline and the other residues of the peptide. We find that *N*_pro,np_/*N*_p_ in the all-*cis* case is more than twice as large as that in the all-*trans* case at the considered temperatures. The result of hs-*cis* is in between the former two cases, and that of hm-*cis* is the lowest. Moreover, *N*_pro,np_/*N*_p_ in the cases of hm-*cis* and all-*cis* remains essentially unchanged with temperature; it decreases in the case of hs-*cis* but increases monotonically in the case of all-*trans* as *T* increases from *T* < *T*_*l*_ to *T* > *T*_*l*_. These results can be explained by the two competing effects: (i) the collapse of the chain results in more intramolecular H-bonds; (ii) the breaking of the β-sheets, if any, leads to fewer intramolecular H-bonds. In the cases of hm-*cis* and all-*cis*, the peptide has nearly no β-sheets, so the value of *N*_pro,np_/*N*_p_ is solely dependent on the compactness of the chain. Since the scattered *trans* isomers in the hm-*cis* case dilute the compactness effects of *cis* isomers, its *N*_pro,np_/*N*_p_ is even smaller than that in the all-*cis* case. In the hs-*cis* case, the number of hydrogen bonds from the block with *cis* isomers is barely changing as a function of *T*. In the other block with *trans* isomers, there are hydrogen bonds formed to stabilize the β-sheets at *T* < *T*_*l*_. This set of hydrogen bonds is gone as *T* > *T*_*l*_ (see Figure S4), which caused the reduction of *N*_pro,np_/*N*_p_ in the collapsed state. In the all-*trans* case, *N*_pro,np_/*N*_p_ slightly increases because the collapse of the chain brings more intramolecular H-bonds at *T* > *T*_*l*_ (the residue separation of involved pairs along the backbone of the peptide can be seen in Figure S10). Note that there are no H-bonds formed among proline residues. Our explanation is also verified by *N*_pp_/*N*; see Figure S10a. *N*_pp_/*N* increases monotonically in the cases of hm-*cis* and all-*cis* but decreases in the cases of all-*trans* and hs-*cis* as *T* increases. Moreover, the study of a semidilute system with several chains indicates that the transition leads to strong chain overlap in the all-*trans* case while the all-*cis* chains also seem to aggregate, however, with a much weaker tendency to interpenetrate. In other words, the interactions between peptides in the all-*cis* case appear to be rather weak compared to these in the all-*trans* case; see Figure S11. Whether this is a kinetic effect or also due to the relatively short chain length studied here needs further studies.

**Figure 5:**
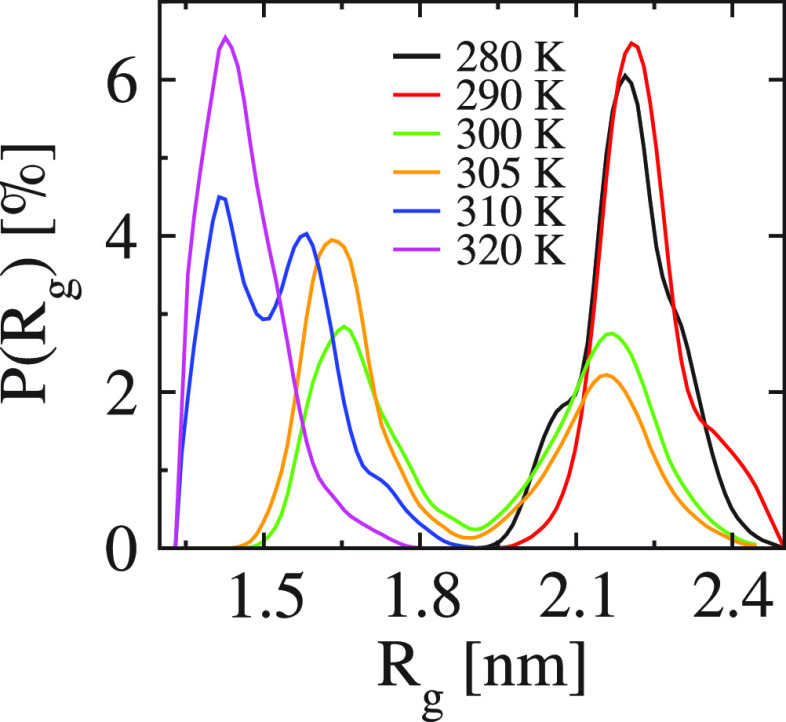
Distribution of *R*_g_ of (VPGVG)_30_ in the all-*trans* case at various temperatures. The bimodal distribution of *R*_g_ is observed in the region 300 K ≤ *T* ≤ 310 K, where the LCST transition occurs.

Finally, we discuss the type of the LCST transition of (VPGVG)_30_ in the all-*trans* case. Theoretically, both the first-order-like and second-order-like phase behavior of macromolecules have been observed.^46^ Polyacetals^47^ are examples of the second-order-like LCST transition. Alternatively, a hysteresis between the heating and cooling procedure around the transition temperature indicates a first-order-like LCST transition. PNIPAm^48^ is one such example. In simulations, a simple way of determining the type of phase transition is to check the distribution of the gyration radius *R*_g_. A bimodal distribution of *R*_g_ near the transition temperature indicates a first-order-like transition, while a unimodal distribution indicates a second-order-like transition. Figure 5 presents the *R*_g_ distribution of (VPGVG)_30_ at different temperatures. The bimodal distribution around its transition temperature *T*_*l*_ clearly demonstrates a first-order-like transition. Clear hysteretic phase behavior in ELP-like IDPs has also been observed experimentally.^49^ Another signature of a first-order-like transition is a broad peak or cusp (with no divergence) of the specific heat *C*_*p*_ around the transition temperature. Figure S12b shows such a peak in agreement with the above conclusion. Furthermore, we find that all four studied cases display the same behavior of *C*_*p*_ in the considered temperature region. While the signatures of a transition for the all-*cis* case were rather small (gradual reduction of H-bonds and weak aggregation), the *C*_*p*_ data support that it experiences an underlying LCST transition, just as the all-*trans* case. A more detailed analysis of the phase diagram will be a subject of future work.

## 4 Conclusions

We have studied the effects of proline isomerization on the phase behavior of an ELP with sequence (VPGVG)_30_ in water. Our results have shown that proline isomerization plays an important role in tuning the conformational behavior of the peptide in the LCST transition, while keeping the transition temperature *T*_*l*_ unchanged. In particular, the peptide exhibited a expanded-to-collapsed transition if all of its proline residues were in the *trans* state, while no such change has been observed if all prolines were in the *cis* state. Moreover, we have found that the number and composition of *cis* proline isomers acted cooperatively in determining the global size and the propensity of secondary structure formation of the peptide. Our work may serve as an inspiration in designing new (bio)polymeric materials and opens a novel direction of regulating the phase behavior of ELPs and other proline-rich peptides.

## Supporting Information Available

Additional simulation details, supporting table, and figures (PDF)
