# Supplementary material for: Proline Isomerization Regulates the Phase Behavior of Elastin-Like Polypeptides in Water
Source: J Phys Chem B. 2021 Aug 23;125(34):9751–6. doi: 10.1021/acs.jpcb.1c04779 (PMC8419842; doi:10.1021/acs.jpcb.1c04779)
Supplement: Supplementary file 1 — jp1c04779_si_001.pdf [file jp1c04779_si_001.pdf]

Supporting Information for Publication: Proline Isomerization Regulates the Phase Behavior of Elastin-Like Polypeptides in Water

Yani Zhao and Kurt Kremer*

Max Planck Institute for Polymer Research, Ackermannweg 10, 55128 Mainz, Germany

E-mail: kremer@mpip-mainz.mpg.de

1 Supporting table and figures

The initial conformations in the all-*trans* case. Figure S1 (a-b) demonstrate the typical initial conformations of $(VPGVG)_{30}$ in the all-*trans* case. One can see that no $\beta-$sheet is formed in these conformations.

ω is not affected by temperatures. In Figure S1 (c-d), we show the distribution of ω dihedral angle of the Val-Pro amide bonds. The average value of ω is $\sim 170°$ or $-15°$ in the *trans* (c) or *cis* (d) case, which remains invariant as T changes.

The gyration radius of $(VPGVG)_{30}$ at $P_{cis} = 0.1$. It has been shown that at ambient temperature up to 12% of Val-Pro bonds form *cis* isomers.[1] To figure out the effects of naturally occurring *cis* isomers on the conformational behavior of the ELP, we consider two additional *cis* compositions with $P_{cis} = 0.1$: ($S_{P_{cis}=0.1}$) *ccctttttt ttttttttt ttttttttt*; ($M_{P_{cis}=0.1}$) *ctttttttt ctttttttt ctttttttt*; The results of R_g for these cases are shown in Figure S2. We find that 10% *cis* content results in 5-13% decease of R_g below the LCST. Since in nature we expect a random isomerization sequence, the

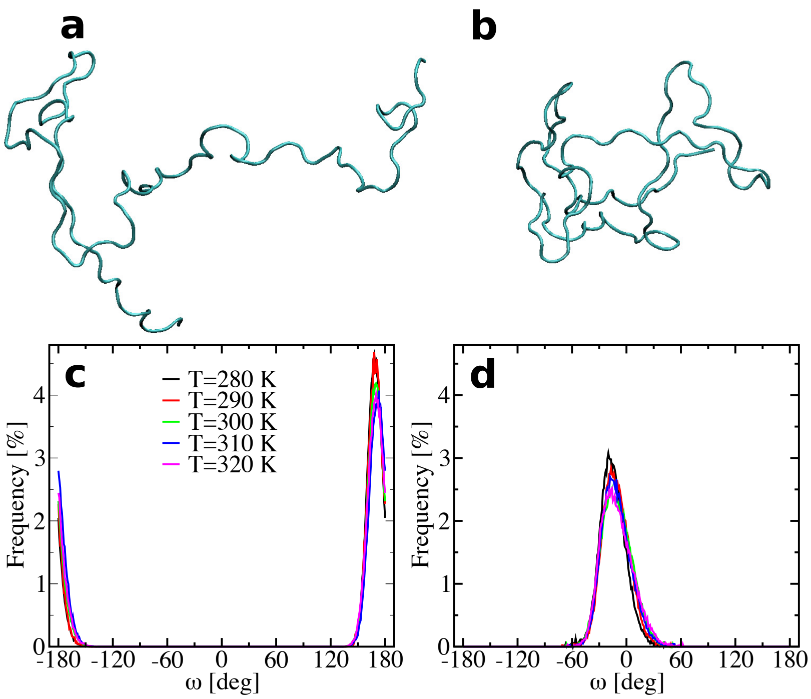

Figure S1: (a-b) Typical examples of the initial conformations in the all-*trans* case. (c-d) The distribution of the ω dihedral angle if proline is in *trans* (c) or *cis* (d) state in (VPGVG)$_{30}$.

$S_{P_{cis}=0.1}$ system describes the weakest while the $M_{P_{cis}=0.1}$ system descirbes the strongest possible naturally occurring effect for (VPGVG)$_{30}$.

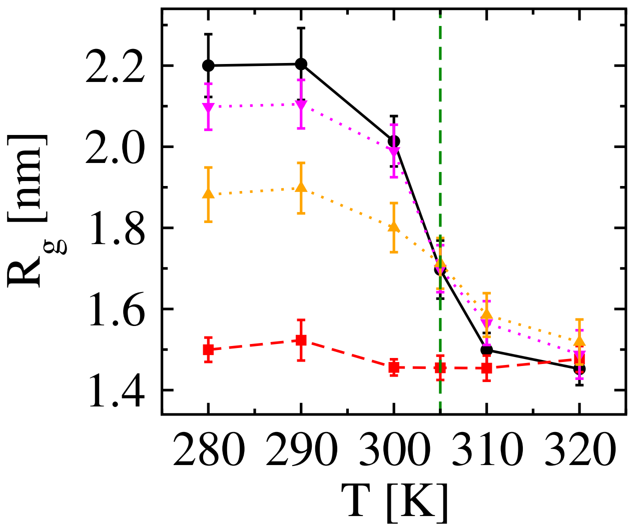

Figure S2: The results of R_g of (VPGVG)$_{30}$ in cases of $S_{P_{cis}=0.1}$ (magenta) and $M_{P_{cis}=0.1}$ (orange). The results in the all-*trans* (black) and all-*cis* (red) cases are also shown as references.

The snapshots of (VPGVG)$_{30}$ in all considered cases. Figure S3 shows the snapshots of

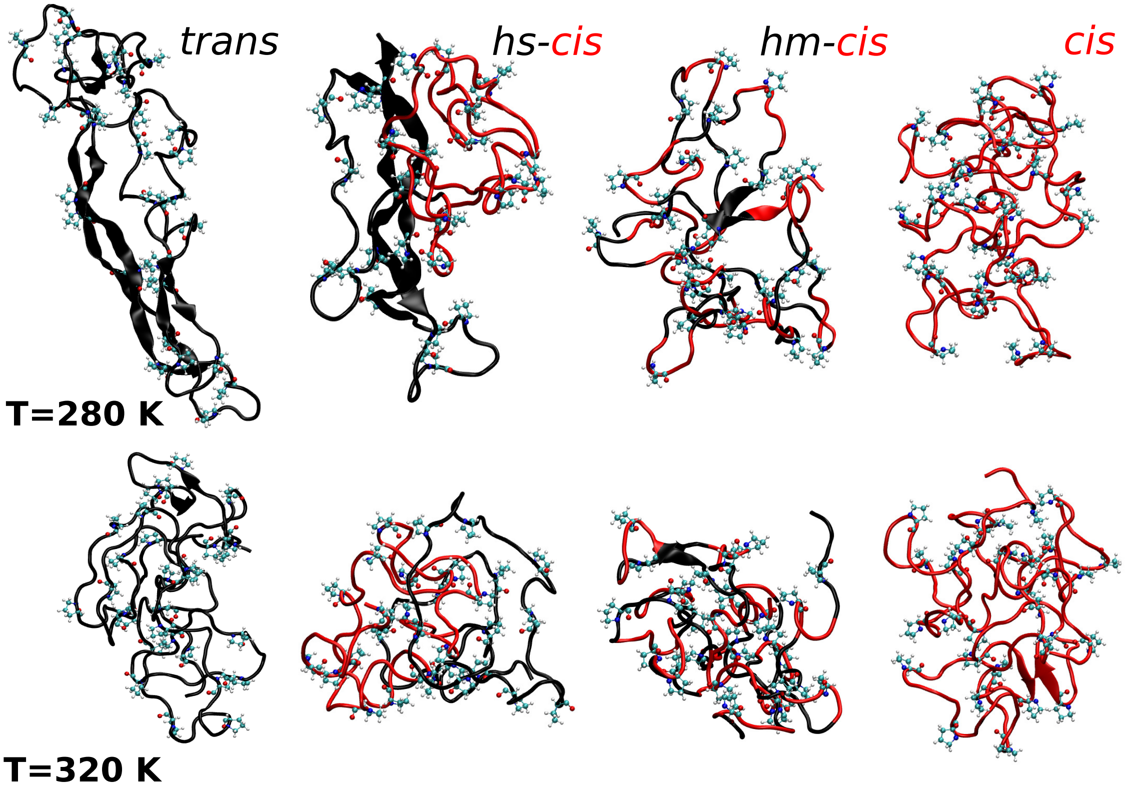

Figure S3: The snapshots of $(VPGVG)_{30}$ in the all-*trans*, hs-*cis*, hm-*cis* and all-*cis* cases in water at $T = 280$ (top panels) and 320 K (bottom panels). The proline residues in all snapshots are demonstrated by the bead-and-stick model.

the ELP at T =280 and 320 K. The region with *trans* isomers is shown in black, while that with *cis* isomers is shown in red. One can see that the number and composition of *cis* isomers work cooperatively in determining the conformation of the peptide.

The $\beta-$sheets formed in the hs-*cis* case are mainly from the region with *trans* isomers. In Figure S4, we separately plot the results of f_β in regions with *trans* or *cis* isomers in the case of hs-*cis*. Clearly, the majority of $\beta-$sheets is from the region with *trans* isomers. The propensity of the $\beta-$sheets was estimated by the DSSP software.[2]

$S(q)$ **of $(VPGVG)_{30}$.** The single-chain backbone structure factor $S(q)$ of the peptide in four considered cases can be found in Figure S5. The scaling law of $S(q)$ is q^{-2}, q^{-3} and q^{-4} if the percentage of the *cis* isomers is $P_{cis} = 1$ (all-*trans*), 0.5 (hs-*cis* and hm-*cis*) and 0.0 (all-*cis*) at $T < T_l = 305$ K, which becomes q^{-4} when $T > T_l$.

Comparison of the radial distribution function $g_{pw}(r)$ at $T < T_l$. In Figure S6 (a), we

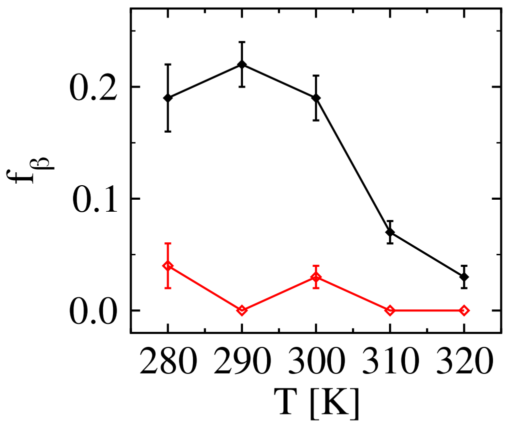

Figure S4: The fraction of $\beta-$sheets f_β formed in the region with *trans* (black) or *cis* (red) isomers in the case of hs-*cis*.

compare the results of $g_{pw}(r)$ at $T = 280$ K. Clearly, the amplitude of the three peaks in $g_{pw}(r)$ satisfies $g_{pw,\text{hm-}cis}(r) < g_{pw,\text{all-}trans}(r) < g_{pw,\text{hs-}cis}(r) < g_{pw,\text{all-}cis}(r)$ at this temperature.

Autocorrelation function of the system. We test the convergence of the trajectories by calculating the autocorrelation function of the end-to-end distance R_e of (VPGVG)$_{30}$, $c(R_e,t) = \frac{\langle R_e(t_0)\cdot R_e(t_0+t)\rangle}{\langle R_e(t_0)\rangle\langle R_e(t_0+t)\rangle}$, where t is the lag time. The results of $c(R_e,t)$ in all considered cases are shown in Figure S6 (b).

The ratios of gyration tensor eigenvalues and SASA. We calculated the gyration tensor $S_{mn} = \frac{1}{2N^2}\sum_i^N\sum_j^N(m_i - m_j)(n_i - n_j)$, where $m,n \in \{x,y,z\}$. The eigenvalues $\lambda_1 \leq \lambda_2 \leq \lambda_3$ of S_{mn} can be used to characterize the sphericity of the chain. The smaller the raio the more spherical the peptide is. The results of λ_3/λ_1 and λ_2/λ_1 are shown in Figure S7 (a), and the corresponding SASA can be found in Figure S7 (b).

Water molecules as H-bond donor or acceptor. In the formed peptide-water H-bonds, water can be either a donor (a hydrogen atom of water is covalently bonded to an oxygen or nitrogen atom of the peptide) or an acceptor (the oxygen atom of water is bonded to a hydrogen atom of the peptide), as it is shown in Figure S8.

Less proline-water H-bonds are formed compared with non-proline residues-water H-bonds. In Figure S9, we show the normalized H-bonds of proline-water $N_{pro,w}/N_p$ and non-proline residues-water $N_{np,w}/(N-N_p)$. We find that less proline-water H-bonds are formed at given T.

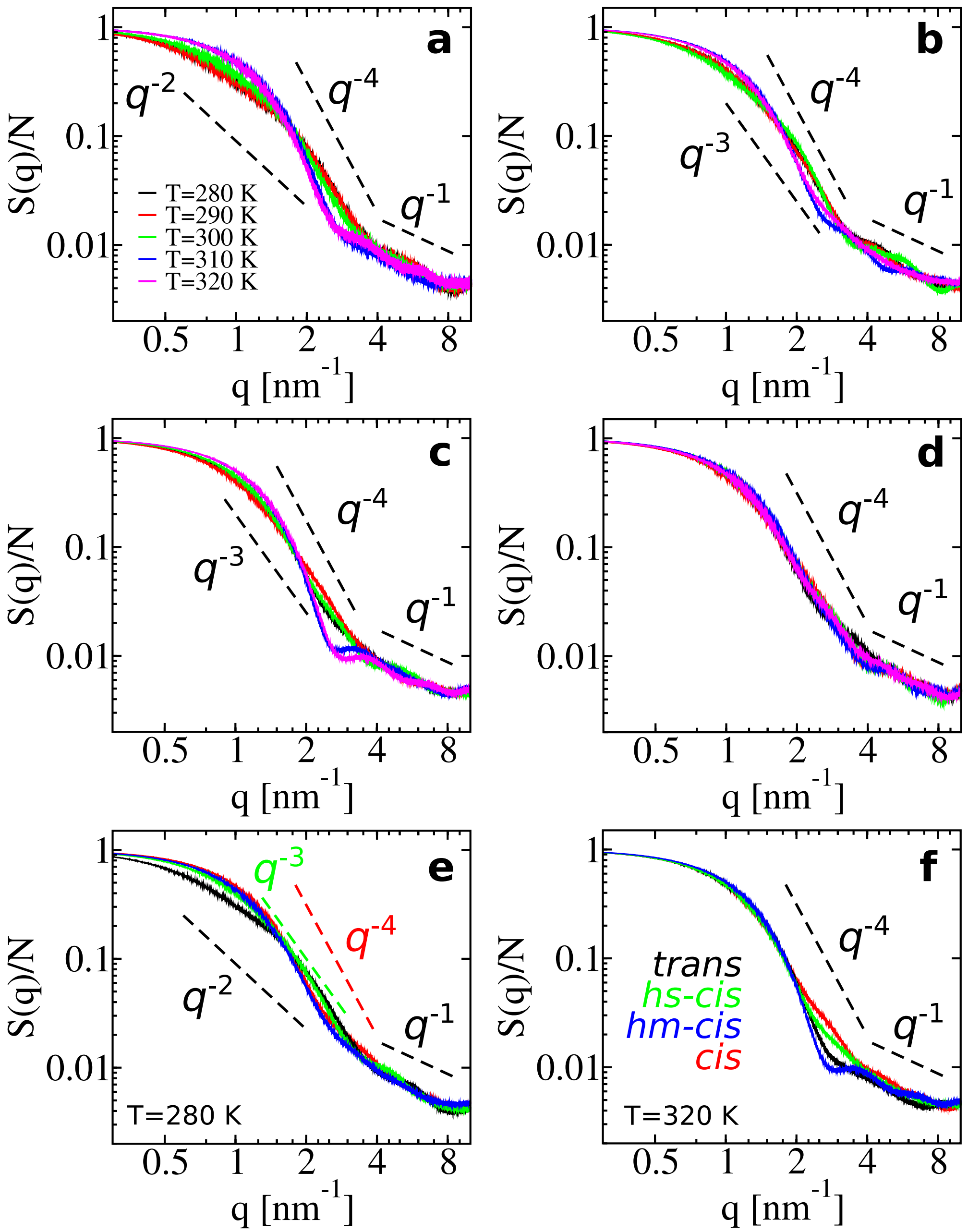

Figure S5: $S(q)$ of $(\text{VPGVG})_{30}$ in the all-*trans* (a), hs-*cis* (b), hm-*cis* (c) and all-*cis* (d) cases at different temperatures in water. The comparisons between the four considered cases at $T = 280$ K and 320 K are shown in panels (e) and (f), respectively.

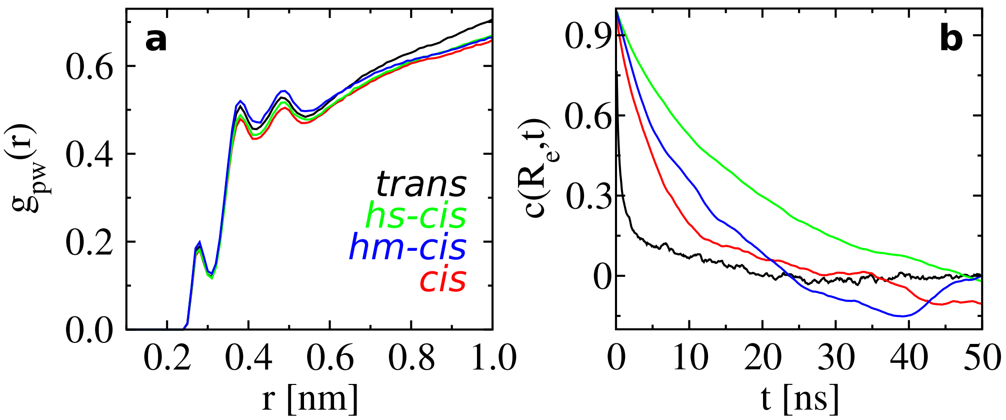

Figure S6: (a) The radial distribution function $g_{pw}(r)$ of (VPGVG)$_{30}$ in the all-*trans* (black), hs-*cis* (green), hm-*cis* (blue) and all-*cis* (red) cases at $T = 280$ K. (b) The autocorrelation function $c(R_e,t)$ of (VPGVG)$_{30}$ in all considered cases at $T = 300$ K.

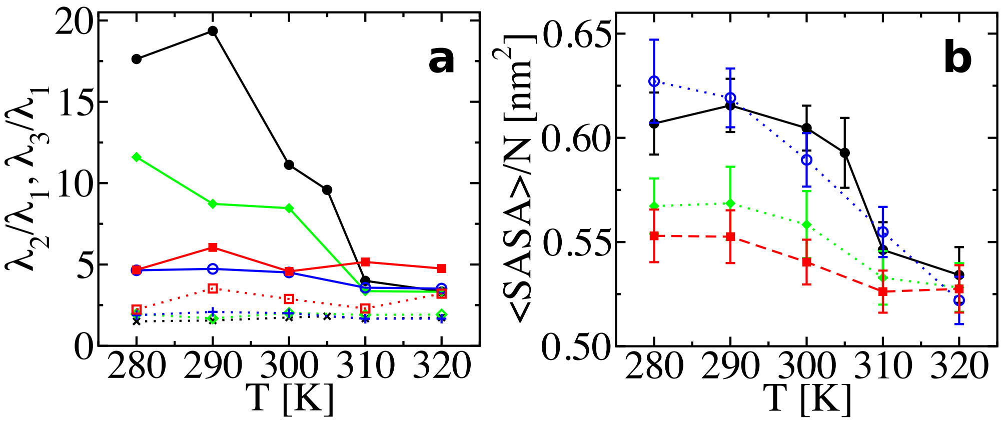

Figure S7: (a) Ratios of the eigenvalues of the gyration tensor: λ_3/λ_1 (solid lines) and λ_2/λ_1 (dashed lines) in the all-*trans* (black), hs-*cis* (green), hm-*cis* (blue) and all-*cis* (red) cases. (b) The normalized solvent-accessible surface area $\langle \mathrm{SASA} \rangle / N$. The color code is the same as panel (a).

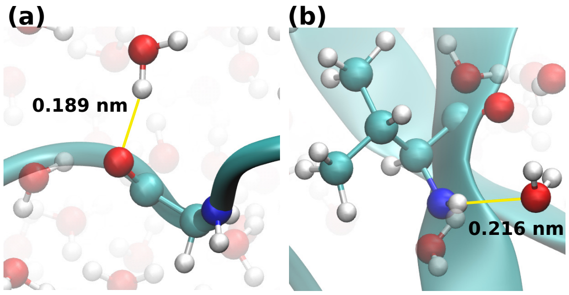

Figure S8: The snapshots of H-bonds formed between the peptide and water molecules, where water can be either H-bonds donors (a) or acceptors (b). The corresponding H-bond length is shown next to the bond.

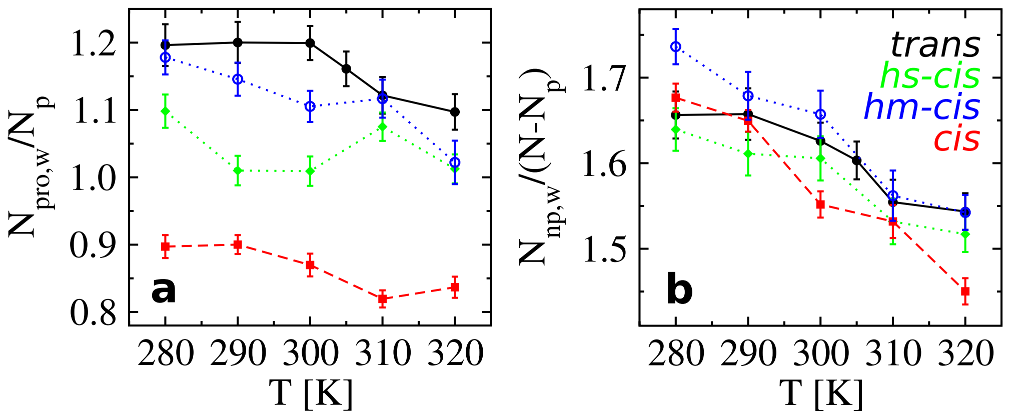

Figure S9: The normalized H-bonds of proline-water $N_{pro,w}/N_p$ (a) and non-proline residues-water $N_{np,w}/(N-N_p)$ (b) in the all-*trans* (black), hs-*cis* (green), hm-*cis* (blue) and all-*cis* (red) cases in water.

Normalized intramolecular H-bonds N_{pp}/N and $N_{np,np}/(N-N_p)$. Figure S10 (a-b) show the results of N_{pp}/N (total intramolecular H-bonds) and $N_{np,np}/(N-N_p)$ (intramolecular H-bonds formed between non-proline residues) as a function of T. We find that as T increases, N_{pp}/N increases monotonically in the cases of hm-*cis* and all-*cis* because of the collapse of the chain, while it decreases in the cases of all-*trans* and hs-*cis* due to the loss of the hydrogen bonds involved in the formation of $\beta-$sheets. $N_{np,np}/(N-N_p)$ has similar temperature trend as N_{pp}/N. Figure S10 (c-d) demonstrate the distribution of the residue separation $|i-j|$ of pair residues i and j involved in the formation of $N_{pro,np}$ (intramolecular H-bonds formed between proline and non-proline residues). One can see that $|i-j|$ in the all-*trans* case is larger than that in the all-*cis* case at $T=280$ K. This is because residues with long distance along the backbone can form H-bonds to stabilize the $\beta-$sheets in the all-*trans* case, while nearly no $\beta-$sheets are observed in the all-*cis* case due to the compactness of the peptide. As the temperature increases to $T=320$ K, the value of $|i-j|$ in the all-*trans* case is alike to the all-*cis* case, because the fraction of $\beta-$sheets is nearly zero and the peptide becomes tightly compacted in both cases at $T>T_l$.

Proline isomerization affects the intermolecular interactions of ELPs. In order to see how proline isomerization affects the intermolecular interactions of ELPs, we considered a system with four $(VPGVG)_{30}$ peptides when all of them are in the all-*trans* (*cis*) case. The peptides are placed in a 10 nm$\times$10 nm$\times$10 nm box with 32 177 water molecules, and thus the system is a semi-dilute

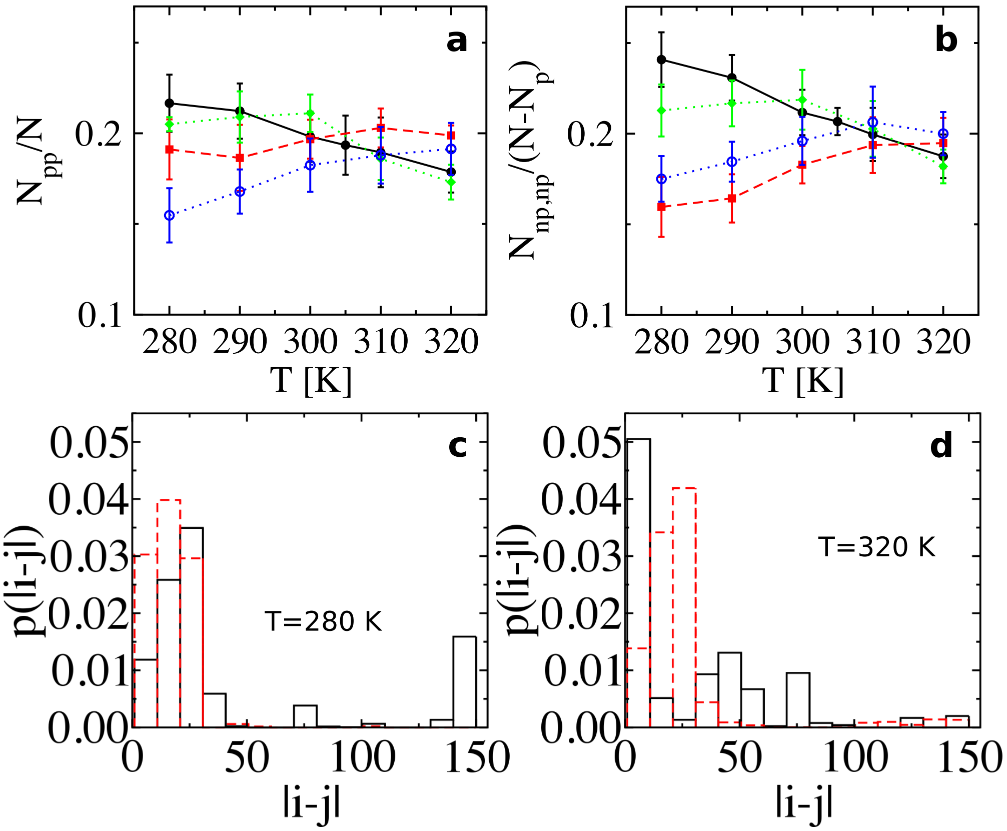

Figure S10: (a) Normalized intramolecular H-bonds N_{pp}/N in the all-*trans* (black), hs-*cis* (green), hm-*cis* (blue) and all-*cis* (red) cases in water. (b) Normalized intramolecular H-bonds $N_{np,np}/(N-N_p)$ formed between non-proline residues. The color code is the same as panel (a). (c) The distribution of the residue separation $|i-j|$ of pair residues i and j involved in the formation of intramolecular H-bonds $N_{pro,np}$ in the all-*trans* (black) and all-*cis* (red) cases at $T = 280$ K ($T < T_l$). (d) The same as panel (c) but for $T = 320$ K ($T > T_l$).

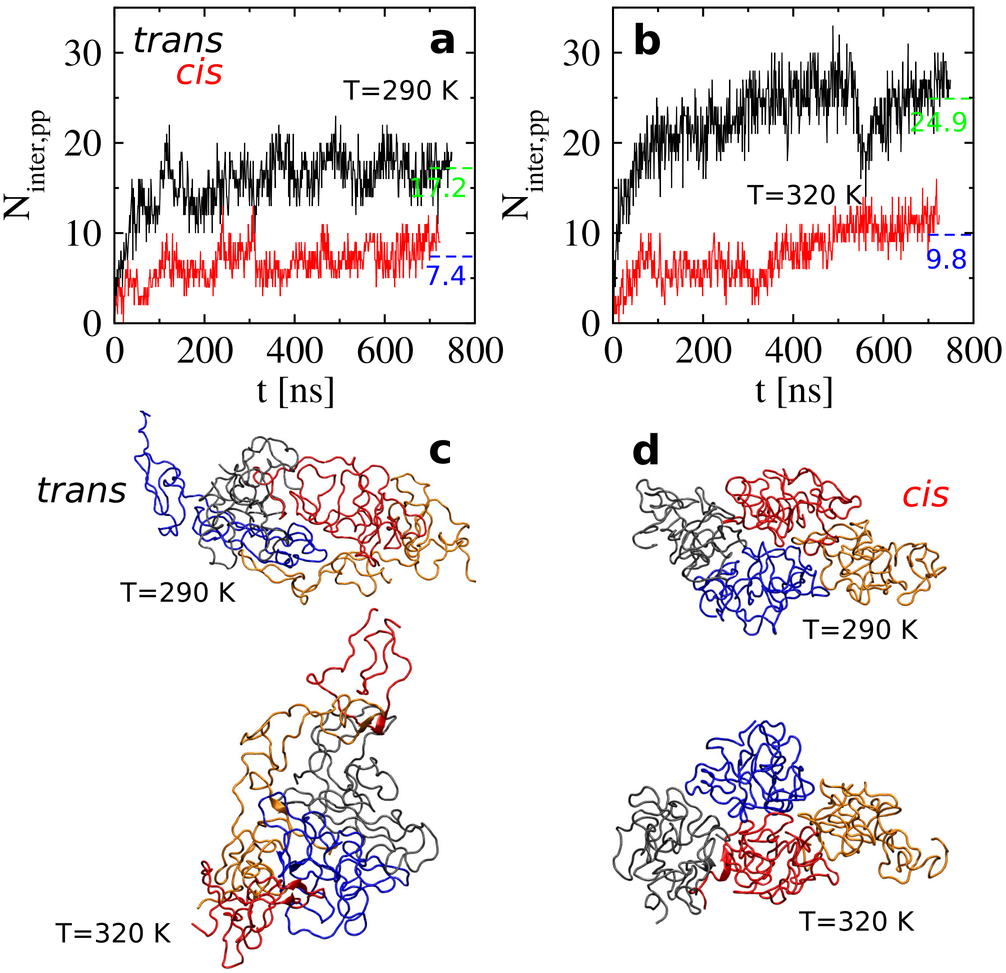

Figure S11: (a) Number of intermolecular H-bonds in the all-*trans* (black) and all-*cis* (red) cases at $T = 290$ K. (b) The same as panel (a) but for $T = 320$ K. (c) Snapshots of the system with four peptides in the all-*trans* case. These peptides are shown in different colors. (d) The same as panel (c) but for the all-*cis* case.

solution with a solute concentration of ∼80 mg/ml. Figure S11 shows the total intermolecular H-bonds $N_{\text{inter},pp}$ among the four peptides if they are in the all-*trans* (*cis*) case. We find that $N_{\text{inter},pp}$ in the all-*trans* case is more than twice larger than that in the all-*cis* case at both $T = 290$ K and 320 K. Moreover, *trans* prolines are more likely to involve in the formation of intermolecular H-bonds than *cis* ones. For example at $T = 320$ K, prolines participate in 22.1% (14.2%) of the intermolecular H-bonds in the all-*trans* (*cis*) case. These data indicate that the compactness of chains with *cis* prolines can form less intermolecular H-bonds. We also observe that $N_{\text{inter},pp}$ increases as T increases from $T < T_l$ to $T > T_l$ in the all-*trans* case, while it remains nearly unchanged in the all-*cis* case. This is because *cis* prolines promote the intramolecular interactions and thus all chains in the system remain tightly self-compact, see Fig. S11 (d). This situation is not changing as T increases, and thus ELPs in the all-*cis* case do only exhibit a weak chain aggregation without significant overlap. It is not clear at the moment, whether this will remain for longer chains and is due to the already collapsed structure below the LCST, or whether there is a large free energy barrier between the compact individual chains and a strongly overlapping regime.

Specific heat of the system. In an isothermal-isobaric (NpT) ensemble, the isobaric heat capacity C_p is defined as:[3]

$$C_p = (\frac{\partial H}{\partial T})_p, \tag{1}$$

where H is the enthalpy of the system.[4] One can calculate C_p by the enthalpy fluctuation formula

$$C_p = \frac{1}{N}\frac{\langle H^2\rangle - \langle H\rangle^2}{k_B\langle T\rangle^2}, \tag{2}$$

where $N = 32177$ is the number of water molecules in our simulations. The enthalpy $H(T)$ of the whole system is calculated by the GROMACS tool gmx energy in the *NPT* ensemble. Moreover, $\langle H^2\rangle$ and $\langle H\rangle$ are the ensemble-average of $H^2(T)$ and $H(T)$. The obtained C_p can be seen in Figure S12. The results of pure water agrees well with the experimental data[5] and the previous simulations for the TIP3P water model.[6] We are aware of the fact that classical models might lead to deviations in C_p compared to experiments due to the lack of correctly dealing with quantum

mechanical constraints on chemical bonds. In the present calculation, this is mostly accounted for by the LINCS algorithm. However, these contributions only would very weakly change over the temperature range discussed here, and thus are not relevant for the maximum in C_p observed here.

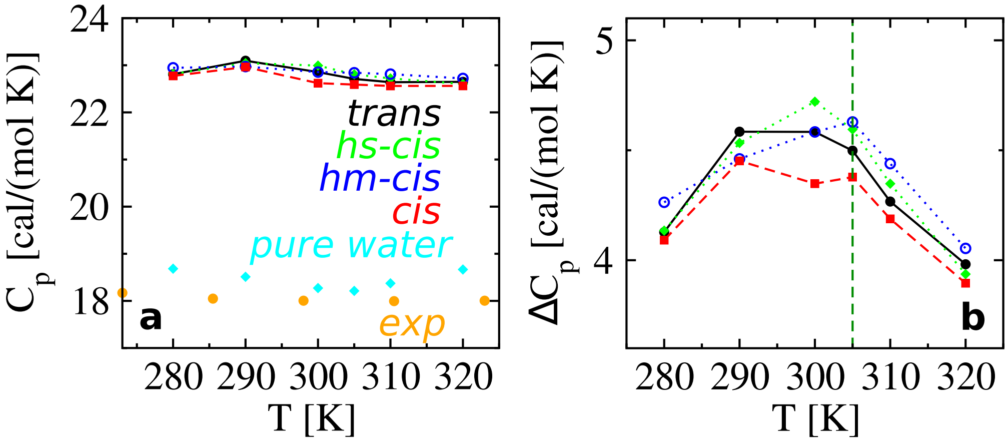

Figure S12: (a) The obtained C_p by using Equation (2) for the whole system in cases of the all-*trans* (black), hs-*cis* (green), hm-*cis* (blue), all-*cis* (red) and pure water (cyan) at $p = 1$ bar. The orange dots are the experimental data of water.[5] (b) The plot of $\Delta C_p = C_{p,i} - C_{p,w}$, where $i \in \{$all-*trans*, hs-*cis*, hm-*cis* and all-*cis*$\}$, and $C_{p,w}$ is the heat capacity of pure water with the same number of water molecules as the whole system at $p = 1$ bar.

Comparison of the shape parameters. The results of the hydrodynamic radius R_h (which is defined as $\frac{1}{R_h} = \frac{1}{N^2}\langle\sum_{ij}\frac{1}{\mathbf{r}_{ij}}\rangle$, where $\mathbf{r}_{ij} = |\mathbf{r}_i - \mathbf{r}_j|$ and $\mathbf{r}_i$ corresponds to the position of the C_α atom of the ith residue along the backbone of $(VPGVG)_{30}$), gyration radius R_g and the end-to-end distance R_e are shown in Table S1.

References

(1) Dugave, C.; Demange, L. Cis-trans isomerization of organic molecules and biomolecules: implications and applications. *Chem. Rev.* **2003**, *103*, 2475–2532.

(2) Kabsch, W.; Sander, C. Dictionary of protein secondary structure: pattern recognition of hydrogen-bonded and geometrical features. *Biopolymers* **1983**, *22*, 2577–2637.

(3) Chaikin, P. M.; Lubensky, T. C. *Principles of Condensed Matter Physics*; Cambridge University Press: Cambridge, 1995.

Table S1: R_h, R_g and R_e of $(VPGVG)_{30}$ in the all-*trans*, hs-*cis*, hm-*cis* and all-*cis* cases at five different temperatures. Please note that the experimental value[7] of R_h for $(VPGVG)_{30}$ is 3.4 nm.

T	280 K	290 K	300 K	310 K	320 K
$R_{h,\text{all-}trans}$ [nm]	3.61±0.10	3.62±0.12	3.43±0.09	3.08±0.08	3.10±0.10
$R_{h,\text{hs-}cis}$ [nm]	3.25±0.11	3.27±0.10	3.37±0.12	3.05±0.09	3.08±0.10
$R_{h,\text{hm-}cis}$ [nm]	3.26±0.06	3.27±0.14	3.16±0.15	3.04±0.10	3.02±0.08
$R_{h,\text{all-}cis}$ [nm]	3.10±0.08	3.12±0.08	3.00±0.06	2.98±0.14	3.07±0.10
$R_{g,\text{all-}trans}$ [nm]	2.20±0.08	2.20±0.09	2.01±0.06	1.50±0.04	1.45±0.04
$R_{g,\text{hs-}cis}$ [nm]	1.81±0.07	1.80±0.08	1.80±0.08	1.49±0.06	1.43±0.07
$R_{g,\text{hm-}cis}$ [nm]	1.71±0.03	1.69±0.03	1.62±0.04	1.48±0.03	1.48±0.03
$R_{g,\text{all-}cis}$ [nm]	1.50±0.02	1.52±0.05	1.46±0.02	1.45±0.04	1.48±0.07
$R_{e,\text{all-}trans}$ [nm]	2.34±0.15	2.26±0.14	2.81±0.16	2.90±0.16	2.95±0.15
$R_{e,\text{hs-}cis}$ [nm]	3.92±0.15	3.81±0.18	3.72±0.19	3.46±0.16	3.48±0.12
$R_{e,\text{hm-}cis}$ [nm]	3.47±0.10	3.44±0.16	3.39±0.12	3.40±0.11	3.43±0.19
$R_{e,\text{all-}cis}$ [nm]	2.53±0.16	2.38±0.18	2.46±0.16	2.65±0.15	2.43±0.12

(4) Horn, H. W.; Swope, W. C.; Pitera, J. W.; Madura, J. D.; Dick, T. J.; Hura, G. L.; Head-Gordon, T. Development of an improved four-site water model for biomolecular simulations: TIP4P-Ew. *J. Chem. Phys.* **2004**, *120*, 9665–9678.

(5) Horn, H. W.; Swope, W. C.; Pitera, J. W.; Madura, J. D.; Dick, T. J.; Hura, G. L.; Head-Gordon, T. The IAPWS formulation 1995 for the thermodynamic properties of ordinary water substance for general and scientific use. *J. Phys. Chem. Ref. Data* **2002**, *31*, 387–535.

(6) Akinkunmi, F. O.; Jahn, D. A.; Giovambattista, N. Effects of temperature on the thermodynamic and dynamical properties of glycerol-water mixtures: A computer simulation study of three different force fields. *J. Phys. Chem. B* **2015**, *119*, 6250–6261.

(7) Fluegel, S.; Fischer, K.; McDaniel, J. R.; Chilkoti, A.; Schmidt, M. Chain stiffness of elastin-like polypeptides. *Biomacromolecules* **2010**, *11*, 3216–3218.
